# Review of "Biomedical Engineering Principles", by A.B. Ritter, S. Reisman and B.B. Michniak

**DOI:** 10.1186/1475-925X-4-63

**Published:** 2005-11-02

**Authors:** Amit Gefen

**Affiliations:** 1 Department of Biomedical Engineering, Faculty of Engineering, Tel Aviv University, Tel Aviv 69978, Israel

Being a young discipline, Biomedical Engineering (BME) offers too few good textbooks for undergraduates. Moreover, in some of the available undergraduate BME textbooks, the levels of mathematics and engineering science were compromised in favor of a more qualitative approach. I was therefore happy to discover that in the recent title by Ritter et al., there is not only in-breadth overview on current BME fields, but also, in-depth analysis of specific selected topics that are supported by appropriate mathematical models, equations, quantitative data charts and tables. This indicates, however, that this book is intended for BME undergraduates at the sophomore, junior or senior years (but not for freshmen). Specifically, to work with this textbook, a student should have already learned calculus, differential and integral equations, and basic engineering courses such as statics, dynamics, solid and fluid mechanics and analog electrical circuits. It should be acknowledged that the authors provide overviews on some of these topics where relevant (e.g. they develop the general equations of continuity and motion before discussing hemodynamic flows in Chapter 3), however, such overviews are too brief to actually be able to introduce students to the theoretical basis. Instead, it appears that they were aimed at refreshing prior knowledge. Hence, instructors may use this book in a BME program to support advanced-year courses in biomechanics (cardiovascular or musculoskeletal) or biomedical signal processing, but not in a first-year Introduction to BME course. Considering the broad scope of this book, however, it is not recommended as a sole textbook in a biomechanics or a biomedical signal processing class. Contrarily, in a non-BME engineering program (e.g. which includes some elective BME advanced-year courses), this title can certainly serve as the major textbook to be used in class.

The book contains 12 chapters that are organized in 3 parts. Part 1 (Chapters 1–4) concerns transport processes, cell physiology and the cardiovascular system. Specifically, Chapter 1 includes an overview of engineering analysis of physiological systems with the aid of models, with a thorough discussion on model validation and parameter estimation. Chapter 2 focuses on the cellular level, and discusses cellular processes such as diffusion, transport, generation of membrane potential and propagation of action potentials. Particular attention has been given to muscle contraction at the microscopic scale, with distinction between processes in cardiac and smooth muscles. Chapter 3 is back at the organ scale, and concerns hemodynamic flows as analyzed by engineering tools, including electrical analogs, models of flows in tubes (steady, pulsatile, and turbulent), and characterization of heart sounds. Chapter 4 is a natural continuation, which extends the basic hemodynamic principles described in the previous chapter to flow, pressure and volume relations in the cardiovascular system. It also contains a comprehensive description of the electrocardiogram (ECG), its method of measurement, and its interpretation (e.g. how to identify arrhythmias, myocardial infraction, arterial and ventricular diseases, etc.). A useful feature in this chapter is mathematical models for the left ventricle and the arterial system with their embodiment in Simulink (MathWorks Co., Natick MA, USA) [1]. A detailed description of heart failure conditions closes this chapter.

The second part of the book is a deep and thorough introduction to biomedical signal processing. It is linked to the previous part on modeling by the concept that ultimately, bioengineering models are compared with and validated against experimental data. Acquisition, processing and reduction of physiological signals are therefore essential bioengineering tools that should be thought at the undergraduate BME level. Accordingly, Chapter 5 presents biomedical signals such as the ECG, the electromyogram (EMG), and the electroencephalogram (EEG). It then describes the Fourier transform and its utility in processing these signals. Practical aspects, such as 60 Hz component rejection are provided in this context. The following Chapter 6 concerns acquisition, sampling, and signal windowing, and discusses typical problems such as selection of a sampling frequency and aliasing. Chapter 7 introduces the reader to common techniques for physiological signal processing such as autoregressive modeling, time-frequency and wavelet analyses. Examples of physiological signal processing follow in Chapter 8. These include heart rate variability analysis, blood pressure variability, body temperature oscillations, EMG and EEG analyses in the frequency domains and particularly, median frequency of the EMG as a measure of muscle fatigue. The second part ends with two chapters on biomechanics: Chapter 9 presents some principles of biomechanics, mostly in whole-body musculoskeletal biomechanics but also in tissue mechanics and thermal regulation, and Chapter 10 details some applications. Applications are given in postural stability, biomechanics of swimming, design of ankle prostheses, human power augmentation devices and exoskeleton prototypes, functional electrical stimulation and movement control, decompression models for divers, prediction of pressure ulcer formation on the skin and analysis of lung sounds in normals and emphysematic patients. This is a broad collection of examples, however, as mentioned earlier, all examples are thoroughly analyzed by means of quantitative engineering tools, including biostatistical analyses and models that are based on empirical data.

The third and last part (which is also the shortest part) of this book is dedicated to tissue engineering. Chapter 11 begins with a historical perspective on the field of tissue engineering, which is followed by the paradigms of tissue engineering, a review of materials used as scaffolds in tissue engineering with particular emphasis on degradable polymers but also some reference to ceramics and composites, and a discussion on biological interactions. The chapter ends with an overview on tissue engineering applications including skin substitutes, cardiovascular components, bone regeneration, tissue-engineered muscles and nerve regeneration. The book closes with Chapter 12 on future trends in BME, which contains the authors' perspective on the future of cardiac care, minimally invasive robotic surgery, the next generation of computed tomography imaging and nanotechnology in BME.

In addition to the thorough quantitative handling of the topics in this book, there are review problems at the end of each chapter that allow a student to rehearse the material covered, however, no solutions are included, and this may be frustrating for some students. One reservation that should be noted is that the quality of artwork in the book is not uniform, and some figures are actually blurred, e.g. in Chapter 6 on signal acquisition and processing. Nevertheless, this is a minor issue and the authors should be commended for their achievement – a good, thorough BME textbook for undergraduates that does not move away from using advanced mathematics or engineering theories when needed. I will certainly use examples from the relevant chapters in my Musculoskeletal Biomechanics and Tissue Biomechanics courses, and will add this book to the recommended literature in these courses.
